# Supplementary figures and images for: Characterisation of NLRP3 pathway-related neuroinflammation in temporal lobe epilepsy
Source: PLoS One. 2022 Aug 16;17(8):e0271995. doi: 10.1371/journal.pone.0271995 (PMC9380933; doi:10.1371/journal.pone.0271995)

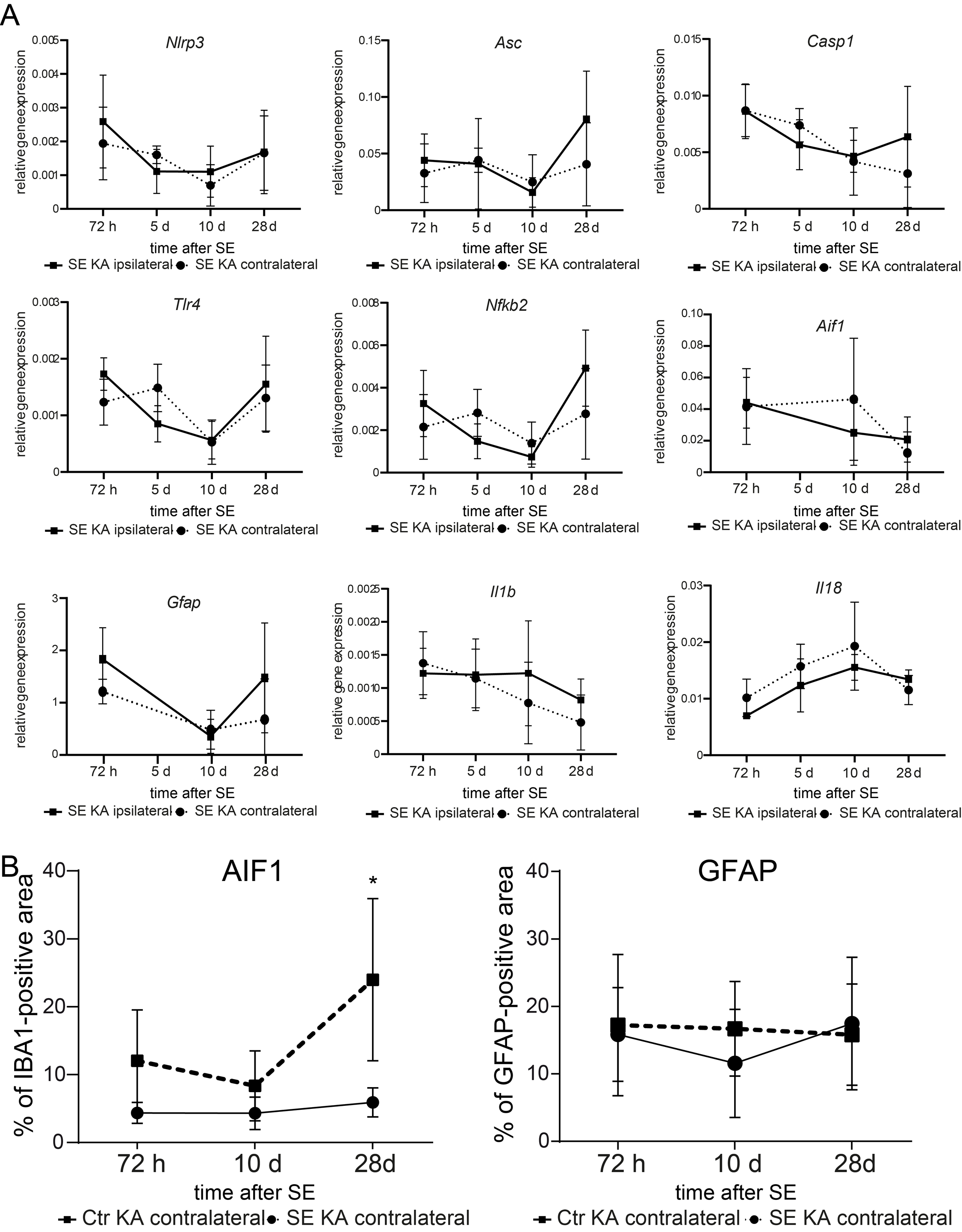

Supplement: S1 Fig — (A) Relative mRNA levels (ΔΔCt method) are analysed 72 h (n ≥ 4), 5 d (n ≥ 5), 10 d (n ≥ 4) and 28 d (n ≥ 6) after kainic acid-induced SE comparing ipsi- and contralateral CA1 with 2way ANOVA followed by Sidak’s post hoc test. The ubiquitously expressed β-actin is used as housekeeping gene. (B) Semi-quantitative analysis of AIF1- or GFAP-positive area reveal nearly no significant changes over time in the contralateral CA1 (all groups n = 3) with 2way ANOVA followed by Sidak’s post hoc test. An increase in microglia (AIF1) was only evident contralaterally in the later model phase (28 days). Asterisks indicate significant differences between groups: *p < 0.05. Detailed statistical values are found in S2 Table. (TIF) [file pone.0271995.s001.tif]

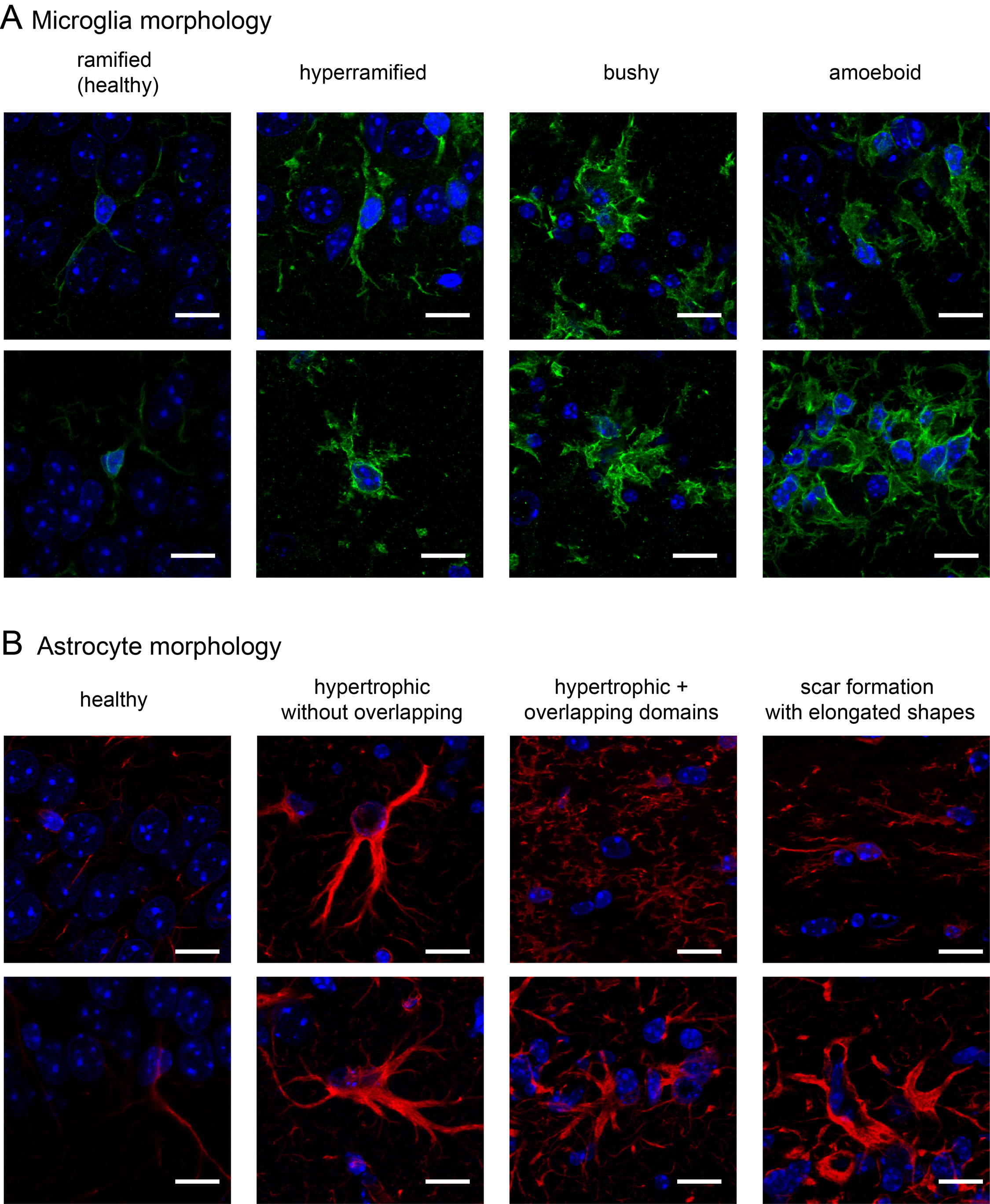

Supplement: S2 Fig — (A) Visualisation of AIF1 positive microglia (green) and (B) GFAP positive astrocytes (red) presenting different shapes of soma and projections depending on the activation status after SE. Scale bars: 20 μm. (TIF) [file pone.0271995.s002.tif]

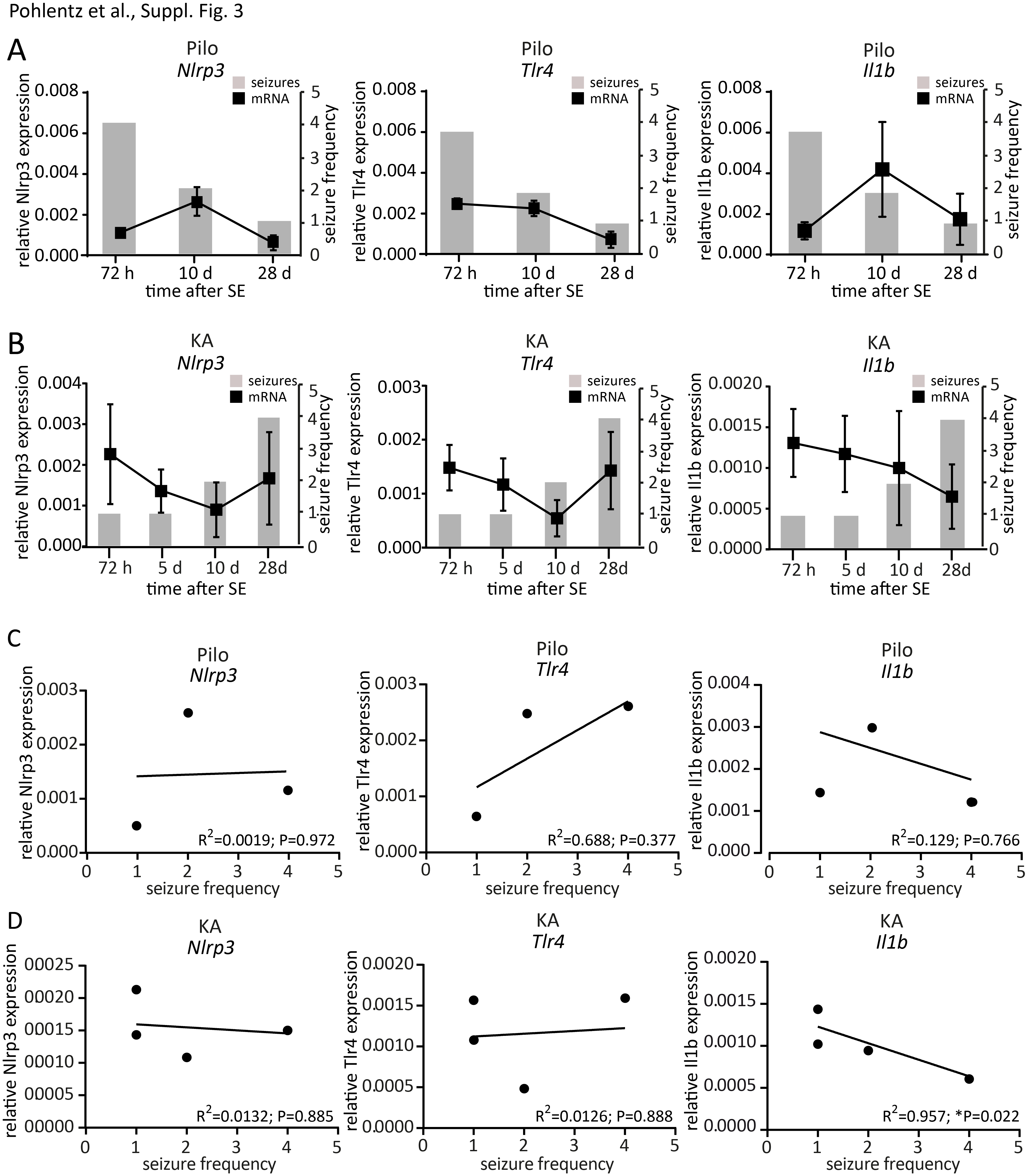

Supplement: S3 Fig — (A, B) Median of the summarized seizure frequency of three consecutive days before mRNA analysis for every animal after (A) pilocarpine (n = 11) and (B) KA-induced SE (n = 21) correlated to mRNA expression level of representative genes. (C, D) Simple regression analysis of seizure frequency and mRNA gene expression of three representative genes analysed after (C) pilocarpine and (D) KA-induced SE. Asterisks indicate significant differences between groups: *p < 0.05. Detailed statistical values are found in S2 Table. (TIF) [file pone.0271995.s003.tif]
