# Supplementary material for: Characterisation of NLRP3 pathway-related neuroinflammation in temporal lobe epilepsy
Source: PLoS One. 2022 Aug 16;17(8):e0271995. doi: 10.1371/journal.pone.0271995 (PMC9380933; doi:10.1371/journal.pone.0271995)
Supplement: S1 Table — LEV = Levetiracetam. Engel class IA: completely seizure free; class IVB: no seizure freedom. (PDF) [file pone.0271995.s004.pdf]

S1 Table. Distribution of clinical parameters with the "lesion associated" and hippocampal sclerosis (HS) patient groups

	Lesion associated	(n)	HS	(n)
Number of patients	34		78	
Gender (male vs. female)	64.7% vs. 35.3%	34	47.4% vs. 52.6%	78
Age at seizure onset (years)	10.7 ± 1.5	34	11.5 ± 1.5	78
Seizure frequency per month	16.5 ± 6.0	34	13.9 ± 2.9	78
Drug therapy (sodium-channel blocker monotherapy vs. LEV combination vs. Non LEV-combination)	17.6% vs. 35.3% vs. 47.1%	34	20.5% vs. 37.2% vs. 42.3%	78
Age at epilepsy surgery (years)	23.0 ± 2.8	34	35 ± 1.7	78
Post-operative outcome (Engel IA vs. Engel IVB)	45.5% vs. 54.5%	33	61.3% vs. 38.7%	75

LEV = Levetiracetam
Engel class IA: completely seizure free; class IVB: no seizure freedom
