# Supplementary material for: Characterisation of NLRP3 pathway-related neuroinflammation in temporal lobe epilepsy
Source: PLoS One. 2022 Aug 16;17(8):e0271995. doi: 10.1371/journal.pone.0271995 (PMC9380933; doi:10.1371/journal.pone.0271995)
Supplement: S2 Table — (PDF) [file pone.0271995.s005.pdf]

S2 Table. Statistical analysis

Fig. 1B: Control vs SE Pilo: 2way ANOVA				
	Group comparison (Control vs. SE)		Time comparison (72h, 10d, 28d)	
	P value	F(DFn, DFd)	P value	F(DFn, DFd)
Nlrp3	**** P<0.0001	F(1,27) = 93.33	**** P<0.0001	F(2,27) = 32.01
Asc	**** P<0.0001	F(1,28) = 87.85	**** P<0.0001	F(2,28) = 20.93
Casp1	**** P<0.0001	F(1,27) = 25.63	*** P=0.0002	F(2,27) = 12.30
Tlr4	**** P<0.0001	F(1,28) = 155.7	**** P<0.0001	F(2,28) = 29.54
NfkB2	**** P<0.0001	F(1,28) = 90.29	**** P<0.0001	F(2,28) = 27.38
Sidak's post hoc test: *p < 0.05, **p < 0.01, ***p < 0.001, ****p < 0.0001				
Fig. 1C: Control vs SE KA: 2way ANOVA				
	Group comparison (Control vs. SE)		Time comparison (72h, 5d, 10d, 28d)	
	P value	F(DFn, DFd)	P value	F(DFn, DFd)
Nlrp3	**** P<0.0001	F(1,82) = 53.87	** P=0.0034	F(3,82) = 4.917
Asc	**** P<0.0001	F(1,70) = 32.22	P=0.0771	F(3,70) = 2.378
Casp1	**** P<0.0001	F(1,74) = 55.27	*** P=0.0042	F(3,74) = 4.785
Tlr4	**** P<0.0001	F(1,82) = 105.9	*** P=0.001	F(3,82) = 5.964
NfkB2	**** P<0.0001	F(1,70) = 34.54	** P=0.0029	F(3,70) = 5.138
Sidak's post hoc test: *p < 0.05, **p < 0.01, ***p < 0.001, ****p < 0.0001				
Fig. 2A/B: Control vs SE Pilo: 2way ANOVA				
	Group comparison (Control vs. SE)		Time comparison (72h, 10d, 28d)	
	P value	F(DFn, DFd)	P value	F(DFn, DFd)
Il1b	*** P=0.0003	F(1,23) = 18.36	* P=0.0204	F(2,23) = 4.629
Il18	P=0.97	F(1,27) = 0.00198	P=0.0771	F(2,27) = 1.745
Sidak's post hoc test: *p < 0.05, **p < 0.01, ***p < 0.001, ****p < 0.0001				
Fig. 3A/B: Control vs SE KA: 2way ANOVA				
	Group comparison (Control vs. SE)		Time comparison (72h, 5d, 10d, 28d)	
	P value	F(DFn, DFd)	P value	F(DFn, DFd)
Il1b	**** P<0.0001	F(1,65) = 77.05	** P=0.0016	F(3,65) = 5.707
Il18	P=0.3261	F(1,69) = 0.978	***P=0.0007	F(3,69) = 6.441
Sidak's post hoc test: *p < 0.05, **p < 0.01, ***p < 0.001, ****p < 0.0001				
Fig. 4A: Control vs SE Pilo: 2way ANOVA				
	Group comparison (Control vs. SE)		Time comparison (72h, 10d, 28d)	
	P value	F(DFn, DFd)	P value	F(DFn, DFd)
Aif1	**** P<0.0001	F(1,26) = 158.5	**** P<0.0001	F(2,26) = 30.03
Gfap	**** P<0.0001	F(1,24) = 123.5	** P=0.0039	F(2,24) = 7.041
Sidak's post hoc test: *p < 0.05, **p < 0.01, ***p < 0.001, ****p < 0.0001				
Fig. 4B: Control vs SE Pilo: 2way ANOVA				
	Group comparison (Control vs. SE)		Time comparison (72h, 10d, 28d)	
	P value	F(DFn, DFd)	P value	F(DFn, DFd)
Aif1	**** P<0.0001	F (1, 12) = 54,66	***P=0.0003	F (2, 12) = 16,69
Gfap	**** P<0.0001	F (1, 12) = 64,95	P=0.1181	F (2, 12) = 2,566
Sidak's post hoc test: *p < 0.05, **p < 0.01, ***p < 0.001, ****p < 0.0001				
Fig. 5A: Control vs SE KA: 2way ANOVA				
	Group comparison (Control vs. SE)		Time comparison (72h, 5d, 10d, 28d)	
	P value	F(DFn, DFd)	P value	F(DFn, DFd)
Aif1	**** P<0.0001	F(1,52) = 35.35	*P=0.0123	F(2,52) = 4.8
Gfap	**** P<0.0001	F(1,57) = 37.51	** P=0.0089	F(2,57) = 5.134
Sidak's post hoc test: *p < 0.05, **p < 0.01, ***p < 0.001, ****p < 0.0001				
Fig. 5B: Control ipsi vs SE ipsi KA: 2way ANOVA				
	Group comparison (Control vs. SE)		Time comparison (72h, 5d, 10d, 28d)	
	P value	F(DFn, DFd)	P value	F(DFn, DFd)
Aif1	**** P<0.0001	F (1, 12) = 46,54	P=0.0533	F (2, 12) = 3,781

Gfap	**** P<0.0001	F (1, 12) = 57,57	P=0.0648	F (2, 12) = 3,467

Sidak's post hoc test: *p < 0.05, **p < 0.01, ***p < 0.001, ****p < 0.0001

Fig. 6D: lesion- vs. HS-TLE: Mann Whitney U-Test

	Group comparison (lesion vs. HS)
	P value
Gfap	**** P<0.0001
Aif1	P=0.3107
Il1B	P=0.2449
Il18	P=0.4736
Nlrp3	P=0.448
Casp1	P=0.9511
Tlr4	*P=0.0183
Nfkb2	P=0.2449

Fig. Suppl. 1: ipsilateral SE vs. contralateral SE: 2way ANOVA

	Group comparison (ipsilateral SE KA vs. contralateral SE KA)		Time comparison (72h, 5d, 10d, 28d)	
	P value	F(DFn, DFd)	P value	F(DFn, DFd)
Nlrp3	P=0.649	F (1, 38) = 0.2109	* P=0.0442	F (3, 38) = 2.963
Asc	P=0.377	F (1, 34) = 0.8015	* P=0.0455	F (3, 34) = 2.970
Casp1	P=0.484	F (1, 34) = 0.2561	* P=0.0096	F (3, 34) = 4.459
Tlr4	P=0.834	F (1, 38) = 0.0446	** P=0.0024	F (3, 38) = 5.734
NfkB2	P=0.550	F (1, 34) = 0.3651	** P=0.0013	F (3, 34) = 6.564
Aif1	P=0.674	F (1, 26) = 0.1815	* P=0.0218	F (2, 26) = 4.451
Gfap	P=0.087	F (1, 29) = 3.138	** P=0.0065	F (2, 29) = 6.013
Il1b	P=0.293	F (1, 31) = 1,145	* P=0.0285	F (3, 31) = 3,446
Il18	P=0.092	F (1, 30) = 3.033	*** P=0.0007	F (3, 30) = 7.411

Fig. Suppl. 2: correlation seizure frequency vs. gene expression: simple linear regression

	Pilo			KA		
	P value	F	R squared	P value	F	R squared
Nlrp3	P=0.9720	F = 0.001939	0.00194	P=0.8849	F=0.02685	0.0132
Tlr4	P=0.3773	F = 2.205	0.688	P=0.8878	F=0.02552	0.0126
Il1b	P=0.7660	F=0.148	0.129	*P=0.0218	F=44.34	0.957

Additional statistics on correlation between seizure frequency vs. gene expression: simple linear regression

	Pilo			KA		
	P value	F	R squared	P value	F	R squared
Asc	P=0.7203	F=0.221	0.181	P=0.4280	F=0.973	0.327
Casp1	P=0.96	F=0.00394	0.00394	P=0.3116	F=1.80	0.474
NfkB2	P=0.9882	F=0.000345	0.000345	P=0.4217	F=1.00	0.334
Aif1	P=0.817	F=0.0874	0.0803	P=0.0774	F=67.0	0.985
Gfap	P=0.7424	F=0.183	0.155	P=0.8465	F=0.0605	0.057
Il18	P=0.0989	F=40.8	0.976	P=0.8728	F=0.0329	0.016
