# Supplementary material for: Characterisation of NLRP3 pathway-related neuroinflammation in temporal lobe epilepsy
Source: PLoS One. 2022 Aug 16;17(8):e0271995. doi: 10.1371/journal.pone.0271995 (PMC9380933; doi:10.1371/journal.pone.0271995)
Supplement: Attachment — Submitted filename: 01_Response_letter_Final.pdf [file pone.0271995.s006.pdf]

Responses to the individual referees' comments on the manuscript entitled "Characterisation of NLRP3 pathway-related neuroinflammation in temporal lobe epilepsy" (Manuscript ID: PONE-D-21-35199)

Editor

Point 1: The authors are urged to have the manuscript given a hard copyedit for syntax and grammar.

Response: The entire revised document was read by a native speaker and corrected for syntax, grammar and spelling.

Reviewer 1.

The individual points of the reviewer were addressed in detail as follows:

Point 1: models of epilepsy are not well characterized: more information is needed on the frequency and severity of seizures, the percentage of animals that do not respond or die, and the progression of the epileptic process. These data could be very useful to the authors for a more in-depth discussion of the expression patterns of inflammasome components at different time points in epileptogenesis.

It should be clarified for example whether 10 days after chemoconvulsant treatment, the animals are in the latency period (without recurrent spontaneous seizures) or in chronic epilepsy. In the discussion, the authors mention that the two animal models (pg 18- second paragraph) have a similar seizure frequency in the chronic period, but they report no confirmatory data.

Response: We thank the referee for this comment. The reviewer is correct in the notion that we did not report on extensive details of the epilepsy phenotype of the models used in our study. We have limited this information, since it would be a pure repetition of published data. In fact, both epilepsy models used in our study are very well established and there is an extensive body of literature on their characterization. We believe that a key novelty aspect of the present manuscript is given by the expression patterns of inflammasome components; we discuss those with regard to these published data. The definition of a latency ("silent") intervals in the epileptogenesis models may represent an own independent topic (Becker, 2018; Levesque et al., 2021). In fact, 24/7 EEG deep electrode measurements accompanied with video monitoring reveals ongoing seizure activity even in the days immediately after chemoconvulsant treatment (Mazzuferi et al., 2012; Pitsch et al., 2017). Considering these aspects, we hope that the referee is in line with our preference to refer to time points after SE induction in days rather than in model 'stages', which may insinuate already interpretational aspects that are not directly related to the topic of this manuscript. These considerations have been included in the manuscript in a condense fashion and we have added two additional references (Bedner et al., 2015; Curia et al., 2008) that also describe key data on the seizure pattern of both models in great detail (p. 20, l. 26 – p. 21, l. 1).

Point 2: general clinical information regarding TLE patients should be provided: adding age, sex, time of epilepsy diagnosis, seizure frequency, drug treatment, the authors could present a more complete characterization of tissue samples.

Response: According the reviewers' suggestion, we have now added a table with the general clinical information in the Supplement (Suppl. table 1).

Distribution of clinical parameters with the "lesion associated" and hippocampal sclerosis (HS) patient groups				
	Lesion associated	(n)	HS	(n)
Number of patients	34		78	
Gender (male vs. female)	64.7% vs. 35.3%	34	47.4% vs. 52.6%	78
Age at seizure onset (years)	10.7 ± 1.5	34	11.5 ± 1.5	78
Seizure frequency per month	16.5 ± 6.0	34	13.9 ± 2.9	78
Drug therapy (sodium-channel blocker monotherapy vs. LEV combination vs. Non LEV-combination)	17.6% vs. 35.3% vs. 47.1%	34	20.5% vs. 37.2% vs. 42.3%	78
Age at epilepsy surgery (years)	23.0 ± 2.8	34	35 ± 1.7	78
Post-operative outcome (Engel IA vs. Engel IVB)	45.5% vs. 54.5%	33	61.3% vs. 38.7%	75
Engel class IA: completely seizure free; class IVB: no seizure freedom				

Hippocampal biopsies from patients with chronic pharmacoresistant mesial TLE were used for the present analysis (Wiebe et al., 2001). In all patients, preoperative examination with a combination of non-invasive and invasive procedures revealed that the seizures originated in the mesial temporal lobe (Kral et al., 2002). Surgical resection of the hippocampus was clinically indicated in all cases because of pharmacoresistance. Hippocampal tissue samples were available for neuropathologic studies for each case included in the present study. The hippocampal sclerosis (HS) group was clearly characterized by segmental neuronal cell loss and concomitant astrogliosis and microglial activation. The hippocampi in the control group showed no segmental neuronal cell loss neuropathologically but had astrogliosis and microglial activation and were therefore consistent with lesions such as cortical dysplasia or epilepsy-associated tumors. In each case, the diagnosis was made by an experienced neuropathologist (AJB) according to international criteria (Becker et al., 2003; Blümcke et al., 2007). We added this additional information in the revised manuscript (p. 9, l. 17- p10, l. 1).

Point 3: some findings should be better addressed and discussed: Why does IL1beta protein expression increase at 72 h post SE in the pilocarpine model whereas mRNA at 10 days? Furthermore, the authors observed IL1beta signals in a clustered group of neurons. Does this evidence find confirmation in the literature?

Response: At 72 h after pilocarpine-induced SE, we see IL1-beta protein expression clustered only in a small number of neurons. To quantify mRNA expression, we analyzed a large proportion of the dorsal CA1 region. Thus, the locally increased IL1-beta expression 72 h post SE may have been below the detection limit due to a dilution effect. Clustered expression of IL1 beta in neurons associated with TLE has been shown previously in rats after electrically induced SE within the stimulated CA3 region (De Simoni et al., 2000)**.** Segmental neuronal cell loss in CA1 is a common hallmark after pilocarpine-induced SE in chronic rodents (Becker et al., 2008; Mello et al., 1993). However, using TUNEL or silver staining, neuronal cell death was detected in a clustered group of neurons in wildtype mice already 2 days after pilocarpine-induced SE in the CA1 region (Becker et al., 2008; Covolan and Mello, 2000). SE induced by systemic administration of pilocarpine is generally regarded to induce primarily indirect neuronal damage by hyperexcitation, and thus to a clustered pattern of damage. In contrast, local application of kainic acid above the CA1 region preferentially acts by widespread excitotoxic effects on neurons (Vezzani et al., 1999), which then induces a diffuse inflammation and IL1 beta expression in the CA1 area that is severe enough to be detectable by mRNA quantification. We have included relevant aspects in the manuscript (pp. 18, ll. 11-24).

Point 4: In the kainate model, GFAP mRNA and protein show a different time course: why at 10 days GFAP mRNA does not increase while many GFAP positive cells are present on ipsilateral tissue slices?

Response: For analyzing mRNA levels, we used the relative quantification ΔΔct-method (Becker et al., 2008; Fink et al., 1998). Expression levels of mRNA transcripts of the gene of interest are normalised to a reference gene, which is not affected by the experiment. For this purpose, we used the ubiquitously expressed gene beta actin (*Actb*), which is known to be stably expressed in the time course of induced epilepsy models (Chen et al., 2001; Marques et al., 2013; Pernot et al., 2010). The assessment of mRNA expression of *Gfap* is relative to the expression level of the reference gene *Actb* and is therefore also dependent on the change in total cell mass. Therefore, increased protein expression as evident in immunohistochemical analyses may not directly be translatable into mRNA quantitative measures, since e.g. dilution effects due to mixtures of cell input may interfere. In addition, there may be a time lag between mRNA generation and protein expression as the translational process is highly variable and complex (Ingolia et al., 2011). Post-translational modification, such as phosphorylation, which are known to allow the protein to function, also take different length of times (Ramazi and Zahiri, 2021). Thus, it is a common phenomenon that mRNA and protein expression for a particular gene of interest are not immediately interchangeable.

The variance between two groups was analyzed as a function of time using a 2-way ANOVA. This resulted in a p-value of 0.0089 for time variance and a $p<0.0001$ for group variance indicating that both groups are strongly significant different with respect to the analyzed time points and also the time point has a major impact. As pairwise comparisons in post-hoc tests are based on fewer cases than analysis of variance, this reduces the sensitivity. Therefore, the 10d time point does not appear statistically significant using the less sensitive post-hoc test, although it does show an increase when considered individually, but the difference in the other time points is also accounted for in this type of calculation.

Therefore, a combination of both methods, immunohistochemistry and quantitative mRNA analysis, is essential in order to make a meaningful statement of the disease pattern. We have included this additional information in the revised manuscript (p. 20, ll. 18-23).

Point 5: page 6 in the induction of chronic epilepsy by systematic injection of pilocarpine, please specify which animals were used.

Response: The same animals (male C57Bl6/N mice; Charles River; ~60 days old, weight ≥ 20 g) were used for both induction models. We have included this additional information in the revised manuscript (p. 6, ll. 21-22).

Point 6: Data are represented with different graphical forms: the best layout is the one as scatterplot and should be used also in fig.1, 2 and 3.

Response: We thank the reviewer for this helpful comment. According to his/her suggestion we have now adjusted the relevant figures.

Reviewer 2.

We thank the referee for commending that 'The manuscript is well written and the data are interesting and consistent'. We particularly thank this referee for the constructive suggestion to change the presentation of our present data to a regular research article format. We feel that the manuscript has substantially benefitted from following this advice.

Point 1: Please, avoid complex/confuse sentences (e.g. the last paragraph of the introduction section should be rewritten).

Response: We thank the reviewer for this comment. We corrected the referring sentence in the revised version of the manuscript (p. 3, ll. 15-19). We also looked through the entire text and simplified all the longer sentences.

Point 2: The statistical tests applied and their goals should be described in the material and methods section. The suppl. material only presents the analysis results, but not describe the statistical methods properly.

Response: We now included the information on the applied statistical tests in the Material and Methods section (p. 11, ll. 10-16).

Point 3: The authors affirm that it was observed an increased number of microglial and astroglial cells (main text and Figs. 4 and 5). However, they did not perform a morphometric analysis (e.g. fluorescence intensity of AIF1 and GFAP or positive cell count) to confirm this statement.

Response: We addressed the Reviewer's idea and performed further studies to quantify the morphology of microglia and astrocytes. As suggested, we now included a quantitative analysis by measuring the positively-stained area of AIF1 and GFAP to confirm our statement. Semi-quantitative analysis now underlines the picture of activation of GFAP-positive astrocytes and IBA1-positive microglia seen in the histological overview. In both models, the hippocampal formation shows an increased level of activated cells analyzed by measuring the area of AIF1- or GFAP-positive cells. The new data can now be found in Fig 4B and 5B.

Point 4: According to the authors, "additional immunolabeling against NLRP3 also revealed a similar expression pattern in both TLE pathologies". How was this expression pattern evaluated (morphological and/or morphometric analysis)? Please, include this information in the materials and methods section.

Response: We added the information that the expression pattern was evaluated by morphological analysis (p. 11, ll. 4-5 and p. 16, l. 10-11).

Point 5: The authors affirm that "In the chronic phase, densely packed and highly activated microglia and astrocytes along with increased Gfap and Aif1 mRNA level are found in the KA-model". Although the number of microglia seemed increased in KA-model compared to control group at 28 days, the Aif1 mRNA level was not. Once again, the morphometric analysis of AIF1 expression should be useful to complement the mRNA findings.

Response: Please see our answer to Point 3.

References

Becker, A.J., et al., 2003. Correlated stage- and subfield-associated hippocampal gene expression patterns in experimental and human temporal lobe epilepsy. Eur J Neurosci. 18, 2792-802.

Becker, A.J., et al., 2008. Transcriptional upregulation of Cav3.2 mediates epileptogenesis in the pilocarpine model of epilepsy. J Neurosci. 28, 13341-53.

Becker, A.J., 2018. Review: Animal models of acquired epilepsy: insights into mechanisms of human epileptogenesis. Neuropathol Appl Neurobiol. 44, 112-129.

Bedner, P., et al., 2015. Astrocyte uncoupling as a cause of human temporal lobe epilepsy. Brain. 138, 1208-22.

Blümcke, I., et al., 2007. A new clinico-pathological classification system for mesial temporal sclerosis. Acta Neuropathol. 113, 235-44.

Chen, J., et al., 2001. Activity-induced expression of common reference genes in individual cns neurons. Lab Invest. 81, 913-6.

Covolan, L., Mello, L.E., 2000. Temporal profile of neuronal injury following pilocarpine or kainic acid-induced status epilepticus. Epilepsy Res. 39, 133-52.

Curia, G., et al., 2008. The pilocarpine model of temporal lobe epilepsy. J Neurosci Methods. 172, 143-57.

De Simoni, M.G., et al., 2000. 1 Eur J Neurosci. 12, 2623-33.

Fink, L., et al., 1998. Real-time quantitative RT-PCR after laser-assisted cell picking. Nat Med. 4, 1329-33.

Ingolia, N.T., Lareau, L.F., Weissman, J.S., 2011. Ribosome profiling of mouse embryonic stem cells reveals the complexity and dynamics of mammalian proteomes. Cell. 147**,** 789-802.

Kral, T., et al., 2002. Preoperative evaluation for epilepsy surgery (Bonn Algorithm). Zentralbl Neurochir. 63**,** 106-10.

Levesque, M., et al., 2021. The pilocarpine model of mesial temporal lobe epilepsy: Over one decade later, with more rodent species and new investigative approaches. Neurosci Biobehav Rev. 130**,** 274-291.

Marques, T.E., et al., 2013. Validation of suitable reference genes for expression studies in different pilocarpine-induced models of mesial temporal lobe epilepsy. PLoS One. 8**,** e71892.

Mazzuferi, M., et al., 2012. Rapid epileptogenesis in the mouse pilocarpine model: video-EEG, pharmacokinetic and histopathological characterization. Exp Neurol. 238**,** 156-67.

Mello, L.E., et al., 1993. Circuit mechanisms of seizures in the pilocarpine model of chronic epilepsy: cell loss and mossy fiber sprouting. Epilepsia. 34**,** 985-95.

Pernot, F., et al., 2010. Selection of reference genes for real-time quantitative reverse transcription-polymerase chain reaction in hippocampal structure in a murine model of temporal lobe epilepsy with focal seizures. J Neurosci Res. 88**,** 1000-8.

Pitsch, J., et al., 2017. Circadian clustering of spontaneous epileptic seizures emerges after pilocarpine-induced status epilepticus. Epilepsia. 58**,** 1-13.

Ramazi, S., Zahiri, J., 2021. Posttranslational modifications in proteins: resources, tools and prediction methods. Database (Oxford). 2021.

Vezzani, A., et al., 1999. Interleukin-1beta immunoreactivity and microglia are enhanced in the rat hippocampus by focal kainate application: functional evidence for enhancement of electrographic seizures. J Neurosci. 19**,** 5054-65.

Wiebe, S., et al., 2001. A randomized, controlled trial of surgery for temporal-lobe epilepsy. N Engl J Med. 345**,** 311-8.
