# Supplementary material for: Characterisation of NLRP3 pathway-related neuroinflammation in temporal lobe epilepsy
Source: PLoS One. 2022 Aug 16;17(8):e0271995. doi: 10.1371/journal.pone.0271995 (PMC9380933; doi:10.1371/journal.pone.0271995)
Supplement: Attachment — Submitted filename: 01_Response letter II.pdf [file pone.0271995.s007.pdf]

Responses to the individual referees' comments on the manuscript entitled "Characterisation of NLRP3 pathway-related neuroinflammation in temporal lobe epilepsy" (Manuscript ID: PONE-D-21-35199)

Reviewer 1.

The individual points of the reviewer were addressed in detail as follows:

Point 1: *The study and correlation of seizures with the dynamics of NLRP3-dependent transcripts and proteins would be of great interest.*

Response: According to the referee's suggestions, we have added highly detailed phenotypic seizure data to the study and calculated the correlation between seizure frequency and gene expression in both models for molecules with representative expression profile changes over the time course of the epilepsy models. A direct correlation between neuronal hyperexcitability (seizure frequency) and gene expression was observed for Il1b in the KA model. Other NLRP3-related genes under study did not show similar expression changes (Representative calculation see S3 Fig and S2 Table). Direct comparability of seizure frequency, mRNA, and protein expression may be affected by time intervals between mRNA generation and protein expression in a gene-dependent manner, as the translation process is highly variable and complex (Ingolia et al., 2011).

For correlation analysis we used the median of the summarized seizure frequency of three consecutive days before mRNA analysis for every animal (day 3: seizure sum of day 1-3; day 5: sum of day 3-5; day10: seizure sum of day 8-10; day 28: sum of day 26-28). The median seizure frequency is now found in Suppl. Fig. 3A correlated to representative genes (NLRP3, TLR4, Il1b). Correlation of these three representative genes is presented in Suppl. Fig. 3B. The statistical analysis of the correlation for all genes under study is found in Suppl. Table 2.

Point 2: *In addition, in one part of the Discussion (p.21 l. 6-12) there is a paragraph with a hypothesis linking seizure frequency to neuroinflammation. Hypothesis that could be supported and discussed extensively with the authors' findings.*

Response: The referee addresses an important point. Our present data suggest that robust positive correlations between expression of the inflammation associated genes under study and seizure frequencies are not evident for these molecules. Thus, our study does not support immediate conclusions such as seizure activity induced inflammation or vice versa. However, given time delays in such interfering mechanisms, we can also not rule out that an interplay between seizure activity and levels of innate inflammation exist in the models under study. To study this more in detail will require selectively interfering functionally with individual molecules under study. However, such approaches clearly go beyond the framework of the present study. We discuss this issue in the revised manuscript (p. 21, l. 22 – p. 22, l. 4).

References

Becker, A.J., et al., 2003. Correlated stage- and subfield-associated hippocampal gene expression patterns in experimental and human temporal lobe epilepsy. Eur J Neurosci. 18, 2792-802.

Becker, A.J., et al., 2008. Transcriptional upregulation of Cav3.2 mediates epileptogenesis in the pilocarpine model of epilepsy. J Neurosci. 28, 13341-53.

Becker, A.J., 2018. Review: Animal models of acquired epilepsy: insights into mechanisms of human epileptogenesis. Neuropathol Appl Neurobiol. 44, 112-129.

Bedner, P., et al., 2015. Astrocyte uncoupling as a cause of human temporal lobe epilepsy. Brain. 138, 1208-22.

Blümcke, I., et al., 2007. A new clinico-pathological classification system for mesial temporal sclerosis. Acta Neuropathol. 113, 235-44.

Chen, J., et al., 2001. Activity-induced expression of common reference genes in individual cns neurons. Lab Invest. 81**,** 913-6.
Covolan, L., Mello, L.E., 2000. Temporal profile of neuronal injury following pilocarpine or kainic acid-induced status epilepticus. Epilepsy Res. 39**,** 133-52.
Curia, G., et al., 2008. The pilocarpine model of temporal lobe epilepsy. J Neurosci Methods. 172**,** 143-57.
De Simoni, M.G., et al., 2000. 1 Eur J Neurosci. 12**,** 2623-33.
Fink, L., et al., 1998. Real-time quantitative RT-PCR after laser-assisted cell picking. Nat Med. 4**,** 1329-33.
Ingolia, N.T., Lareau, L.F., Weissman, J.S., 2011. Ribosome profiling of mouse embryonic stem cells reveals the complexity and dynamics of mammalian proteomes. Cell. 147**,** 789-802.
Kral, T., et al., 2002. Preoperative evaluation for epilepsy surgery (Bonn Algorithm). Zentralbl Neurochir. 63**,** 106-10.
Levesque, M., et al., 2021. The pilocarpine model of mesial temporal lobe epilepsy: Over one decade later, with more rodent species and new investigative approaches. Neurosci Biobehav Rev. 130**,** 274-291.
Marques, T.E., et al., 2013. Validation of suitable reference genes for expression studies in different pilocarpine-induced models of mesial temporal lobe epilepsy. PLoS One. 8**,** e71892.
Mazzuferi, M., et al., 2012. Rapid epileptogenesis in the mouse pilocarpine model: video-EEG, pharmacokinetic and histopathological characterization. Exp Neurol. 238**,** 156-67.
Mello, L.E., et al., 1993. Circuit mechanisms of seizures in the pilocarpine model of chronic epilepsy: cell loss and mossy fiber sprouting. Epilepsia. 34**,** 985-95.
Pernot, F., et al., 2010. Selection of reference genes for real-time quantitative reverse transcription-polymerase chain reaction in hippocampal structure in a murine model of temporal lobe epilepsy with focal seizures. J Neurosci Res. 88**,** 1000-8.
Pitsch, J., et al., 2017. Circadian clustering of spontaneous epileptic seizures emerges after pilocarpine-induced status epilepticus. Epilepsia. 58**,** 1-13.
Ramazi, S., Zahiri, J., 2021. Posttranslational modifications in proteins: resources, tools and prediction methods. Database (Oxford). 2021.
Vezzani, A., et al., 1999. Interleukin-1beta immunoreactivity and microglia are enhanced in the rat hippocampus by focal kainate application: functional evidence for enhancement of electrographic seizures. J Neurosci. 19**,** 5054-65.
Wiebe, S., et al., 2001. A randomized, controlled trial of surgery for temporal-lobe epilepsy. N Engl J Med. 345**,** 311-8.
